# EuDiS - A comprehensive database of the seed dispersal syndromes of the European flora

**DOI:** 10.3897/BDJ.11.e104079

**Published:** 2023-07-11

**Authors:** Pablo Vargas, Ruben Heleno, José M. Costa

**Affiliations:** 1 Department of Biodiversity and Conservation, Real Jardín Botánico de Madrid – CSIC, Madrid, Spain Department of Biodiversity and Conservation, Real Jardín Botánico de Madrid – CSIC Madrid Spain; 2 Centre for Functional Ecology, Associate Laboratory TERRA, Department of Life Sciences, University of Coimbra, Coimbra, Portugal Centre for Functional Ecology, Associate Laboratory TERRA, Department of Life Sciences, University of Coimbra Coimbra Portugal

**Keywords:** anemochory, ballochory, endozoochory, epizoochory, hoarding, hydrochory, long-distance dispersal, myrmecochory, plant diaspore, seed dispersal traits, short-distance dispersal, thalassochory

## Abstract

**Background:**

Seed dispersal is a critical process in plant colonisation and demography. Fruits and seeds can be transported by several vectors (typically animals, wind and water), which may have exerted strong selective pressures on plant’s morphological traits. The set of traits that favour dispersal by a specific vector have been historically considered as seed dispersal syndromes. As seed dispersal syndromes have a great potential to predict how seeds move (i.e. the relative importance of the standard mechanisms of seed dispersal), they have attracted the attention of naturalists and researchers for centuries. However, given that observations of actual dispersal events and colonisation are seldom reported, there is still much confusion in current studies failing to properly discriminate between seed dispersal syndromes (i.e. sets of traits that favour a particular mechanism) and actual seed dispersal (i.e. the vector that moves a given seed in one dispersal event). This distinction is important because the presence of any seed dispersal syndrome does not preclude the seed being occasionally dispersed by other non-standard mechanisms (i.e. different from the one predicted). Similarly, the absence of seed dispersal syndromes does not prevent seeds from being dispersed. The correct coding of seed dispersal syndromes thus requires a systematic and evolutive, rather than a phenomenological approach. Unfortunately, such approach has rarely been implemented at a community-level and no comprehensive datasets of seed dispersal syndromes are yet available for any entire flora.

**New information:**

This database contains categorisation of the native European flora into eight seed dispersal syndromes. Information for a total of 9,874 species retrieved from the volumes of Flora Europaea were analysed. Earlier versions of this database, which only coded for the presence of four long-distance dispersal syndromes (endozoochorous, epizoochorous, thalassochorous and anemochorous diaspores), were used in four previous studies. Here, we present a fully revised and expanded database, including the presence of four additional short-distance dispersal syndromes (myrmecochorous, vertebrate hoarding, freshwater hydrochorous and ballochorous diaspores), a nomenclatural update for all species and the codification of 416 additional species.

Roughly half (51.3%) of the native European flora produce diaspores without traits clearly associated with facilitating seed dispersal. The other half (48.7%) of the European plant species produces diaspores with some specialised traits associated with seed dispersal, most of which (79.9%) with a potential to facilitate long-distance dispersal events. The most common diaspores are those with anemochorous (23.5%), epizoochorous (8.0%), endozoochorous (7.8%), myrmecochorous (7.2%), thalassochorous (2.3%), freshwater dispersal (2.1%), ballochorous (4.6%) and vertebrate hoarding associated traits (0.2%). Two-thirds (66.3%) of the European shrub and tree species have diaspores with some specialisation for biotic seed dispersal.

## Introduction

Seed dispersal is a key process in plant demography due to its recognised importance for the colonisation of new and disturbed areas, for gene flow within populations and for the maintenance of regional species diversity (Janzen 1971, Howe and Smallwood 1982, Traveset et al. 2014). The plant vegetative or generative organ(s) that is(are) actually dispersed and can originate a new adult plant, usually the seeds or fruits with eventual accessory tissues that may facilitate the dispersive event, is(are) called a diaspore (van der Pijl 1982). Seeds can be dispersed by multiple non-exclusive mechanisms, which are often classified as biotic or abiotic, depending on whether they involve an animal vector or not. When studying the result of the dispersal, seed movement is classified as long- or short-distance dispersal (LDD and SDD, respectively) (Heleno and Vargas 2015, Jordano 2017). Although there is no clear line on what is the minimum distance to consider an event as a long-distance dispersal, it has been generally recognised that there are four seed dispersal mechanisms that can result in long-distance dispersal of diaspores in a biogeographical context, namely: endozochory (i.e. internal dispersal in the animal’s gut), epizoochory (external dispersal on the animal’s integument), thalassochory (dispersal on seawater) and anemochory (dispersal by wind) (Heleno and Vargas 2015). While these mechanisms can disperse diaspores at broad scales, the action of other mechanisms is restricted to dispersal of seeds at relatively shorter distances, i.e. within the same population or area, namely: myrmecochory (i.e. dispersal by ants), vertebrate hoarding (i.e. intentional transport by vertebrate, hoarders), freshwater hydrochory (i.e. transport on freshwater courses) and ballochory (i.e. explosive ejection of seeds from the parent plant) (Ridley 1930, van der Pijl 1982). The relative importance of each of these mechanisms in different regions has long stimulated the interest of naturalists, ecologists and biogeographers, especially in the last two centuries. As a result, many researchers began to pay particular attention to the size and form of plant diaspores and to explore seed dispersal probability (Darwin 1859, Guppy 1917, Ridley 1930, Carlquist 1966, Proctor 1968, van der Pijl 1982).

*An important clarification*: Due to the assumed high correspondence between syndromes and their respective vectors (e.g. wings-wind), the presence of diaspore traits and the actual means of dispersal have been often confused in the botanical and ecological literature. On the one hand, the presence of a given set of traits has been often assumed to be sufficient evidence to infer the vector responsible for dispersal events (e.g. if it has wings, it must have been dispersed by wind). On the other hand, the observation of a given dispersal event has been often assumed as sufficient evidence to classify the plant as having a particular seed dispersal syndrome (e.g. if it was internally dispersed by an animal, it must have endozoochorous traits). Although having particular morphological attributes increases the probability of being dispersed by specific mechanisms (Römermann et al. 2005, Carthey et al. 2016, Arjona et al. 2018), repeated observations that seeds can also be dispersed by non-standard mechanisms (e.g. winged seeds dispersed by animals) (Higgins et al. 2003, Heleno et al. 2011, Costea et al. 2019, Green et al. 2022) lead to the growing recognition that syndromes and vectors/mechanisms are highly interrelated, but independent concepts (Heleno and Vargas 2015).

In recent years, there has been a renewed effort in the compilation of trait databases, including those related to seed dispersal (Kleyer et al. 2008, Sádlo et al. 2018, Tavşanoğlu and Pausas 2018). However, most databases are restricted to small regions, such as an archipelago or a country (Vargas et al. 2012), to a small subset of the species pool (Melendo et al. 2003) or they combine data on dispersal syndromes (i.e. traits) and observed dispersal vectors (e.g. events), hindering independent analyses of either of them (Kleyer et al. 2008). A database on the seed dispersal syndromes of most European flora was initially compiled by Heleno and Vargas (2015) and used in three subsequent studies (Correia et al. 2018, Fristoe et al. 2021, Moyano et al. 2022); however, this was restricted to the four syndromes capable for justifying the colonisation of the Azores from European diaspores (i.e. endozoochorous, epizoochorous, thalassochorous and anemochorous). Here, we present a fully revised and annotated version of that dataset which has been fully curated and complemented by scoring the presence of four additional short-distance dispersal syndromes (myrmechorous, vertebrate hoarding, freshwater hydrochorous and ballochorous traits) for the entire European flora: EuDiS European Dispersal Syndromes.

## General description

### Purpose

The goal of this dataset is to provide information about the presence of seed dispersal syndromes for the native plant species listed in Flora Europaea (Tutin et al. 1980). This database is strictly based on the presence or absence of traits – i.e. syndromes – that might facilitate dispersal by each specific mechanism, independently of whether dispersal by any vector has been observed or not. Therefore, this database codes for the presence or absence of evolutionary traits rather than actual ecological processes.

## Sampling methods

### Sampling description

**Species list**

A comprehensive list of native European spermatophytes (i.e. plants with seeds) was initially compiled from the five volumes of Flora Europaea (Tutin et al. 1980). Nomenclature was then updated according to the Plants of the World Online - POWO (https://powo.science.kew.org/) with package U.Taxonstand 1.1.0 (Zhang and Qian 2023) in R 4.2.1 (R Core Team 2022). The entire list was then thoroughly checked to ensure that the updated binomial correctly matched the binomial from Flora Europaea. This nomenclatural update resulted in the merging of 887 previously-recognised species in Flora Europaea that are now considered synonyms of other currently-accepted species in POWO. While each entry now corresponds to a currently-accepted species in POWO, we also kept the information of the original nomenclature in Flora Europaea to allow backtracking. Finally, 61 taxa that are currently considered hybrids were also removed, resulting in a final list of 9,874 seeding plant species native to Europe. Information on plant growth form was initially retrieved from public information available from TRY database (Kattge et al. 2020) and then manually curated and largely completed, based on Flora Europaea and Flora Iberica (Castroviejo 2021).

**Seed dispersal syndromes**

All species were individually coded for the presence of seed dispersal syndromes according to their diaspore morphological traits. Eight syndromes were considered, four with a potential for facilitating long-distance dispersal events (endozoochorous, epizoochorous, thalassochorous and anemochorous diaspores) and four syndromes for which the effects are restricted to relatively short-distance dispersal events on a biogeographical scale, namely (myrmecochorous, vertebrate hoarding, freshwater hydrochorous and ballochorous diaspores). Species that lack any specific traits that can be clearly associated with a particular dispersal mechanism are considered unspecialised regarding seed dispersal. Coding diaspores as “unspecialised” avoids the speculative exercise of erroneously assigning syndromes, based on purely phenomenological observations. Nevertheless, it is important to note that unspecialised diaspores can be dispersed (even regularly) by one or more seed dispersal mechanisms.

**Long-distance seed dispersal syndromes**:

- Endozoochorous diaspores: those with nutritive tissues that favour their deliberate ingestion by animals, typically frugivores and have the capacity to avoid digestion in the gut. This syndrome corresponds largely to diaspores with a large sarcotesta, aril or a fleshy pericarp (van der Pijl 1982, Howe 1986, Fuentes 1994). Diaspores that do not bear nutritive structures, but can be intentionally or inadvertently ingested (e.g. by large herbivores) were not considered endozoochorous even if some of these seeds can be effectively dispersed (e.g. dry fruits, such as achenes). This matches previous reports that seeds with no nutritive structures can be dispersed internally by animals (endozoochory) even if they do not have endozoochorous traits (Heleno et al. 2011, Green et al. 2023).
- Epizoochorous diaspores: those with hooks, spines, barbs, awns or adhesive substances (i.e. mucilage and resins) that promote their external adhesion to the fur, feathers or any other part of the animals’ body (van der Pijl 1982, Sorensen 1986, Cousens et al. 2008, Kreitschitz et al. 2021). Although the awns of grasses can have other non-exclusive roles, such as assisting seed establishment in the soil, grass fruits were considered to have epizoochorous traits because they can facilitate the attachment to animals (Petersen and Kellogg 2022). Diaspores that do not have any of the above-mentioned morphological structures may also be dispersed by epizoochory (e.g. small seeds embedded in mud); however, these were not considered epizoochorous as that trait cannot be unequivocally associated with the promotion of external dispersal by animals.
- Thalassochorous diaspores: those with waxy substances or low-density tissues, such as corky tissues and air chambers, including hairs, pappus and wings that can promote floatability (Guppy 1917, Cousens et al. 2008). Thalassochorous traits should include the capacity to survive after prolonged immersion in saltwater (Guja et al. 2010). However, because only a small minority of the European flora has been subjected to floatability and germinability tests after saltwater exposure (Esteves et al. 2015), we considered the capacity of adult plants to live on salt-exposed habitats (i.e. coastal plants) as an indication of some capacity to germinate and grow under halophilous conditions (Guppy 1906, Fuster‐Calvo et al. 2021).
- Anemochorous diaspores: those with hairs, tufts of cotton, plumes, pappus, a flattened rim or wings, resulting on a high volume/weight ratio and high air resistance (drag) (Cousens et al. 2008).

**Short-distance seed dispersal syndromes**:

- Myrmecochorous diaspores: small seeds from dehiscent fruits with elaiosomes (caruncles, strophioles and fleshy funicles) (Ridley 1930, Lisci et al. 1996, Gorb and Gorb 2003, Ortega-Olivencia et al. 2021). Seeds that are known to be directly consumed by harvester ants, but lack an elaiosome, have not been considered myrmecochorous even though they can be dispersed by ants (Gorb and Gorb 2003).
- Vertebrate hoarding: diaspores with large fruits or seeds with abundant nutritive reserves frequently stored by vertebrates (mammals and birds) for later consumption (Cousens et al. 2008, Vander Wall 2010, Yi et al. 2015). Many of these seeds can survive and germinate even after partial consumption of the endosperm (Perea et al. 2011, García-Hernández et al. 2023), while others eventually escape ingestion by getting lost, forgotten or by the death of the disperser before consumption (Vander Wall and Beck 2011).
- Freshwater hydrochorous diaspores: those associated with plants that produce diaspores with waxy substances or low-density tissues that promote floatability (air chambers, corky tissues, hairs, pappus or wings) and grow in lakes and riparian environments (Guppy 1906, Ridley 1930, Cousens et al. 2008, Carthey et al. 2016).
- Ballochorous: species that produce dehiscent fruits with explosive mechanisms that eject seeds metres away from the mother plant (Ridley 1930, Cousens et al. 2008). Explosion and fruit dehiscence often result from a rapid release of accumulated tension on plant organs due to desiccation or hydration (Ridley 1930).

**Syndrome coding**

We independently coded the presence of each seed dispersal syndrome for each species of the database. The following steps were performed:

1. Information on diaspore traits was assembled through an exhaustive search in the published literature for information on diaspore description and images. The main source of information was Flora Europaea (Tutin et al. 1980), which was complemented with data from Flora Iberica (Castroviejo 2021), books on seed dispersal and diaspore traits (Ridley 1930, van der Pijl 1982, Gorb and Gorb 2003, Cappers et al. 2006, Bojňanský and Fargašová 2007, Idžojtić 2019) and specific publications searched using Google Scholar. Several online resources were particularly valuable to check diaspore morphology, namely Acta Plantarum (https://www.actaplantarum.org), Pladias – Database of the Czech Flora and Vegetation (www.pladias.cz), Dispersal and Diaspore Database (www.seed-dispersal.info) and Herbarium JACA (http://proyectos.ipe.csic.es/herbario). References for species studied in specific papers are included in EuDiS database.
2. Additional information on diaspore traits was gathered through consultation of herbarium vouchers at the *Real Jardín Botánico de Madrid* (MA) and at the *Herbarium da Universidade de Coimbra* (COI), including a collection of diaspores for nearly 2,000 species stored in COI.
3. To gain a deeper insight into some families, we consulted with taxonomists and other botanists with expertise in some selected plant families (see the Acknowledgements section). In addition, one of the authors (Pablo Vargas) published taxonomic accounts of some genera (*Athamantha*, *Olea*, *Sanicula*, *Saxifraga*, *Seseli*) for Flora Iberica.
4. Finally, many small informal experiments performed by the authors (mostly by Pablo Vargas) in the field (air suspension capacity, flotation and adhesion) were also important for assigning the dispersal syndrome to many species.

## Geographic coverage

### Description

Europe, under the extent defined in Flora Europaea (except the Azores), from the Iberian Peninsula and Ireland eastwards to the Urals and from Svalbard southwards to Sicilia and Crete (Tutin et al. 1980).

## Taxonomic coverage

### Description

EuDiS dataset comprises 9,874 seed plant species, from 141 families, native to Europe (29 gymnosperms and 9,845 angiosperms). The list was initially assembled from Flora Europaea (Tutin et al. 1980) and the nomenclature updated according to the Plants of the World Online (https://powo.science.kew.org).

## Traits coverage

EuDiS database includes the categorisation into eight seed dispersal syndromes: (1) endozoochorous, (2) epizoochorous, (3) thalassochorous, (4) anemochorous, (5) myrmecochorous, (6) vertebrate hoarding, (7) freshwater hydrochorous and (8) ballochorous. The classification of a species under one of these traits is exclusively based on the presence of morphological attributes irrespective of the observation of dispersal events.

## Usage licence

### Usage licence

Other

### IP rights notes

Creative Commons Attribution (CC BY) 4.0 Licence

## Data resources

### Data package title

EuDis - A comprehensive database of the seed dispersal syndromes of the European flora.

### Resource link

https://doi.org/10.6084/m9.figshare.22251028

### Number of data sets

1

### Data set 1.

#### Data set name

EuDis - A comprehensive database of the seed dispersal syndromes of the European flora.

#### Data format

csv

#### Download URL

https://doi.org/10.6084/m9.figshare.22251028

#### Description

The raw data and the list of publications that were used to code the dispersal syndromes can be found in the EuDiS.csv file. The EuDiS datafile has the following structure:

**Data set 1.** 

Column label	Column description
Species_Flora_Europaea	binomial from Flora Europaea.
Species_POWO	binomial currently (as of 2022) accepted according to Plants of the World Online (https://powo.science.kew.org).
Family	botanical family of the respective species according to POWO.
Endozoochorous	coded as “1” when the species has diaspores with traits that facilitate internal dispersal by animals; coded as “0” otherwise.
Epizoochorous	coded as “1” when the species has diaspores with traits that facilitate external dispersal by animals; coded as “0” otherwise.
Thalassochorous	coded as “1” when the species has diaspores with traits that facilitate dispersal by transport on salt water; coded as “0” otherwise.
Anemochorous	coded as “1” when the species has diaspores with traits that facilitate dispersal by wind; coded as “0” otherwise.
Myrmecochorous	coded as “1” when the species has diaspores with traits that facilitate external dispersal by ants; coded as “0” otherwise.
Vertebrate_hoarding	coded as “1” when the species has diaspores with traits that facilitate dispersal by seed hoarding animals; coded as “0” otherwise.
Freshwater Hydrochorous	coded as “1” when the species has diaspores with traits that facilitate dispersal by fresh water; coded as “0” otherwise.
Ballochorous	coded as “1” when the species has traits that facilitate the dispersal by explosive mechanisms; coded as “0” otherwise.
Further_clarification_needed	marks specific syndromes whose classification is still doubtful or based on incomplete information and thus warrant further work: END: endozoochorous, EPI: epizoochorous, THA: thalassochorous, ANE: anemochorous, HYD: freshwater hydrochorous, MYR: myrmecochorous, BAL: ballochorous.
Tree_or_shrub	coded as “1” when the species is a tree or a woody shrub; coded as “0” otherwise (herbaceous plants, climbers and suffruticose chamaephytes).
Synonyms	binomials that were considered independent species in the volumes of Flora Europaea, but are currently considered synonyms of the binomials listed in the field “Species_POWO”.
References	sources used to code the syndromes.

## Additional information

**Figure 1.**
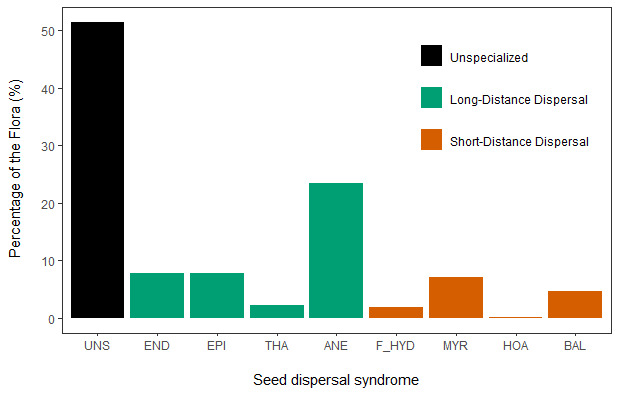
Percentage of the European Flora producing diaspores with (bars in green and red) and without (bar in black) dispersal syndromes. UNS: unspecialised diaspores, END: endozoochorous, EPI: epizoochorous, THA: thalassochorous, ANE: anemochorous, F_HYD: freshwater hydrochorous, MYR: myrmecochorous, HOA: vertebrate hoarding syndrome, BAL: ballochorous.

Of the 9,874 European species assessed, 48.7% produce diaspores with at least one dispersal syndrome (41.7% have only one syndrome, 6.8% have two syndromes and 0.2% have three syndromes) and 51.3% have unspecialised diaspores (Fig. 1). Overall, 9.8% of the species have diaspores with syndromes restricted to SDD, while 38.9% have at least one syndrome with the potential to facilitate LDD events (Table 1). Two thirds (66.3%) of the European shrub and tree species have diaspores with specialisations for at least one form of biotic seed dispersal. The most common syndrome is that associated with anemochorous diaspores (23.5% of all species), followed by the syndromes that promote seed dispersal by animals, almost equally represented in the European flora (7.8% endozoochorous, 8.0% epizoochorous and 7.2% myrmecochorous), except for the vertebrate hoarding syndrome, which is restricted to 0.2% of the flora (genera: *Aesculus*, *Castanea*, *Corylus*, *Fagus*, *Juglans* and *Quercus*) (Fig. 1). Likewise, the percentage of the European flora producing diaspores with traits that can promote seed dispersal by seawater floatation is very similar to that of species with diaspores promoting freshwater dispersal (2.3% and 2.1%, respectively). Finally, 4.6% of the European flora produce dehiscent fruits with ballistic dispersal capacity (i.e. ballochorous) (Fig. 1).

### Maintenance

The current version of the EuDiS database is a baseline work and will be maintained and updated whenever necessary. Future work can involve nomenclature updates, syndrome clarifications/corrections and expanding the species’ list to include invasive species or recently described/recognised species.

## Figures and Tables

**Table 1.** Number and percentage of plant species represented by each seed dispersal syndrome. **LDD**: long-distance dispersal, **SDD**: short-distance dispersal, **Biotic**: the standard dispersal vector is an animal, **Abiotic**: the standard dispersal vector is not an animal.

**Syndrome**	**LDD / SDD**	**Abiotic / Biotic**	**Number of species (%)**
Endozoochorous	LDD	Biotic	774 (7.8%)
Epizoochorous	LDD	Biotic	790 (8.0%)
Thalassochorous	LDD	Abiotic	230 (2.3%)
Anemochorous	LDD	Abiotic	2324 (23.5%)
Myrmecochorous	SDD	Biotic	710 (7.2%)
Vertebrate hoarding	SDD	Biotic	24 (0.2%)
Freshwater hydrochorous	SDD	Abiotic	205 (2.1%)
Ballochorous	SDD	Abiotic	455 (4.6%)
